# REST alleviates neurotoxic prion peptide-induced synaptic abnormalities, neurofibrillary degeneration and neuronal death partially *via* LRP6-mediated Wnt-β-catenin signaling

**DOI:** 10.18632/oncotarget.7640

**Published:** 2016-02-23

**Authors:** Zhiqi Song, Ting Zhu, Xiangmei Zhou, Paul Barrow, Wei Yang, Yongyong Cui, Lifeng Yang, Deming Zhao

**Affiliations:** ^1^ The State Key Laboratories for Agrobiotechnology, Key Laboratory of Animal Epidemiology and Zoonosis, Ministry of Agriculture, National Animal Transmissible Spongiform Encephalopathy Laboratory, College of Veterinary Medicine, China Agricultural University, Beijing, China; ^2^ School of Veterinary Medicine and Science, University of Nottingham, Sutton Bonington, Leicestershire, UK

**Keywords:** RE1-silencing transcription factor, prion diseases, neuroprotective mechanism, the low-density lipoprotein receptor-related protein 6, the Wnt-β-catenin signaling, Pathology Section

## Abstract

Prion diseases are a group of infectious neurodegenerative diseases characterized by multiple neuropathological hallmarks including synaptic damage, spongiform degeneration and neuronal death. The factors and mechanisms that maintain cellular morphological integrity and protect against neurodegeneration in prion diseases are still unclear. Here we report that after stimulation with the neurotoxic PrP106-126 fragment in primary cortical neurons, REST translocates from the cytoplasm to the nucleus and protects neurons from harmful effects of PrP106-126. Overexpression of REST reduces pathological damage and abnormal biochemical alterations of neurons induced by PrP106-126 and maintains neuronal viability by stabilizing the level of pro-survival protein FOXO1 and inhibiting the permeability of the mitochondrial outer membrane, release of cytochrome *c* from mitochondria to cytoplasm and the activation of Capase3. Conversely, knockdown of REST exacerbates morphological damage and inhibits the expression of FOXO1. Additionally, by overexpression or knockdown of LRP6, we further show that LRP6-mediated Wnt-β-catenin signaling partly regulates the expression of REST. Collectively, we demonstrate for the first time novel neuroprotective function of REST in prion diseases and hypothesise that the LRP6-Wnt-β-catenin/REST signaling plays critical and collaborative roles in neuroprotection. This signaling of neuronal survival regulation could be explored as a viable therapeutic target for prion diseases and associated neurodegenerative diseases.

## INTRODUCTION

Prion is a protein-conformation-based infectious agent [1, 2] and causes transmissible spongiform encephalopathies (TSEs) in animals and humans [3]. The neuropathology of prion diseases is characterized by synaptic alterations, spongiform degeneration and neuronal cell death [4] [5, 6]. The causative agent of prion diseases arises from the conformational conversion of cellular prion protein (PrP^C^) to the misfolded form, termed PrP^Sc^, which is pathogenic and neurotoxic, has a higher proportion of β-sheet structure in place of the normal α-helix structure [7] and is protease-resistant [8]. The neurotoxic prion protein fragment 106-126 (PrP106-126) exhibits some of physicochemical and pathogenic properties to PrP^Sc^, in that it forms amyloid fibrils with a high β-sheet content, shows partial proteinase K resistance and is neurotoxic *in vitro* including the ability to cause neuronal cell death and induce proliferation of astrocytes [9-11]. As a widely used model for the *in vitro* study of prion-associated pathological damage, PrP106-126 is used as a material for exploring the molecular mechanism of prion-induced neurogeneration in our research.

The transcription factor REST, a RE1-silencing transcription factor [12], also known as neuron-restrictive silencer factor (NRSF) [13], acts as a transcriptional regulator. REST functions as a hub and with other factors, coordinately regulates multiple aspects of neurogenesis, orchestrates neural differentiation, and preserves the unique neural phenotype [14]. Perturbation of REST expression during embryogenesis causes cellular apoptosis, aberrant differentiation and patterning, and lethality [15]. Additionally, far more than above functions, REST has also been implicated in the pathogenesis and proposed as a therapeutic target of neurodegenerative diseases. Recently, Lu and colleagues demonstrated that REST regulates many genes associated with cell death pathways and Alzheimer's disease (AD) [16]. During normal ageing, REST is induced in part by cell non-autonomous Wnt-β-catenin signaling and localized in the nucleus as a neuroprotective factor [16]. The Wnt-β-catenin signaling pathway has long been associated with the modulation of neurogenesis, dendritic morphogenesis, and synaptic function [17] [18, 19]. More recently, this signaling has been implicated in neurodegenerative disorders such as autism [20], schizophrenia [21] and AD [19]. Previous reports have demonstrated that the Wnt signaling antagonist, Dickkopf-related protein 1 (DKK-1) binds to and inhibits the low-density lipoprotein receptor-related protein 6 (LRP6) in cooperation with the kremen receptor thereby suppressing the Wnt-β-catenin signaling [22]. LRP6 is an essential coreceptor for the canonical Wnt pathway, which is an indispensable element of maintaining synaptic integrity and neuronal viability in AD [23]. Despite these implications, the role and the associated molecular regulatory mechanisms of REST in prion diseases is poorly understood. We thus investigated the function of REST in PrP106-126-induced neuropathology in primary cultured cortical neurons (PCCN) and examined potential regulatory mechanism that function upstream of REST in the LRP6-mediated Wnt-β-catenin signaling.

Herein, we show a novel neuroprotective role for REST in PrP106-126-induced cellular morphological changes and neuronal death *via* increased expression of postsynaptic protein, stabilized level of pro-survival protein and suppressed pro-apoptotic protein. Moreover, we also demonstrate that the LRP6-Wnt-β-catenin signaling partially regulates the expression of REST and cooperatively maintains the morphological integrity and viability of neurons exposed to PrP106-126, thus suggesting REST and this novel signaling pathway could be a particularly attractive therapeutic target of prion diseases.

## RESULTS

### PrP106-126 induces neuropathological changes in PCCN

**Figure 1:**
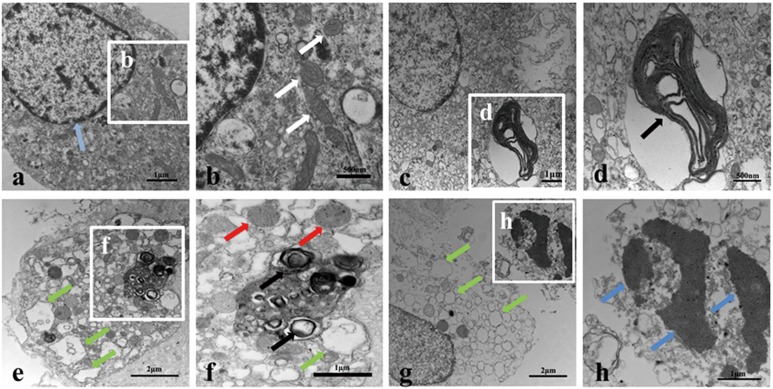
PrP106-126 induces pathological - structural changes in primary cultured cortical neurons (PCCN) **a.**-**h.** Transmission electron microscopy reveals morphological changes of organelles in PCCN exposed to PrP106-126 for 24h. Negative control neurons are treated with PBS showed normal mitochondria (white arrow) and nucleus ultrastructure (light blue arrows) (a, b). PrP106-126-treated neurons (c-h) shows various sizes of myelin figures (black arrows) (c, d); mitochondrial swelling (red arrows)(f); a large number of vacuoles or swollen and disorganized smooth endoplasmic reticulum (green arrows) (e-g); nuclear fragmentation and nuclear condensation (blue arrows)(h).

The classical neuropathological characteristics of transmissible spongiform encephalopathies (TSEs) are synaptic alterations, spongiform change and neuronal cell death [24, 25]. PrP106-126 has been used as a model molecule for studying PrP^Sc^ pathological damage and neurotoxicity, but PrP106-126-induced ultrastructural pathology of PCCN has not been well described. To investigate the neuropathological damage of prion diseases, we observed the morphological changes of PCCN exposed to PrP106-126 by transmission electron microscopy (TEM). Cells without any treatment showed normal organelle ultrastructures (Figure 1a, 1b). By contrast, after exposure to PrP106-126 (200μm) for 24h, the PCCN showed ultrastructural pathology similar to typical “spongiform vacuoles” in TSEs [25]. A large number of vacuoles of various sizes and swollen and disorganized smooth endoplasmic reticulum (SER) appeared in the cytoplasm (Figure 1c and 1g). Occasionally, some myelin figures or concentric lamella structures and autophagosomes or autophagolysosomes were visible as well (Figure 1d and 1f). Dystrophic neurites contained abundant vesicles with a range of sizes and shapes of vacuoles (Figure 1e). Mitochondria were swollen and showed vacuoles and fractured cristae (Figure 1d-1g). Nuclear fragments and condensed chromatin were released into the cytoplasm (Figure 1h).

Consistent with previous researches, we confirmed that PrP106-126 peptide induced mitochondrial cytochrome c release and caspase 3 activation, increased Bcl-2-associated X protein (BAX) and reduced B-cell lymphoma protein 2 (Bcl-2) [26] [27] in a time-dependent manner (S1). Furthermore, PrP106-126-stimulated primary neuronal death was verified by TUNEL (Figure 4d) and Annexin V-FITC/PI (Figure 4k) apoptosis assay. Compared with the results in the control group, the number of apoptotic cells in the PrP106-126-treated group was increased by 31.7% and cell viability was decreased by 42.4%, *P < 0.01*). Our observations proved that PrP106-126 is a valid model for researching PrP^Sc^ neuropathological damage and neurotoxicity.

### PrP106-126 induced expression and translocation of REST to the nucleus

REST has been investigated extensively as a repressor of neuronal genes during embryonic development. Studies have shown that REST is involved in psychiatric and neurological diseases, especially in AD, Huntington's disease and epilepsy [14]. Moreover, extensive evidence demonstrates that prolonged stimulation with various agents, such as 4-aminopyridine (4AP) [28] and amyloid β42 (Aβ42) induce REST expression while loss of nuclear REST in AD is associated with epigenetic derepression of potentially pathogenic genes [16]. We therefore wondered whether REST is activated in PCCN exposed to PrP106-126, or REST merely serves as a cell substrate.

**Figure 2:**
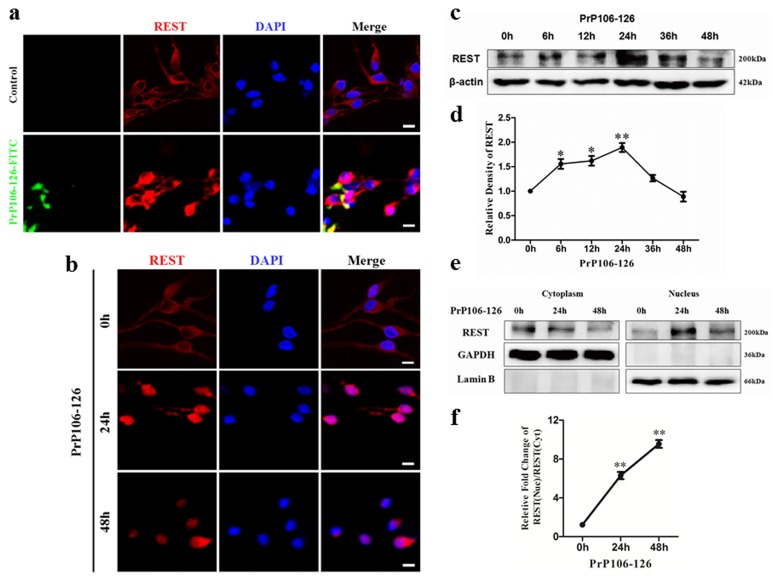
PrP106-126 induces REST expression and translocation from cytoplasm to nucleus **a.**, **b.** Immunofluorescence microscopy reveals translocation of REST from the cytoplasm to nucleus in PCCN exposed to PRP106-126. PCCN are incubated in PBS or FITC-labeled PrP106-126 for 24h (a) or in PBS or PrP106-126 for 24h or 48h (b). Left panel displays the addition of PBS or FITC-labeled PrP106-126(green) in Scale bars = 10μm. c,d Immunoblotting of PCCN cell lysate after treatment with PrP106-126, showing increased REST expression for 24 h before recovery to the baseline level at 48 h. Immunoblotting density of total REST protein is normalized to β-actin and shown as the ratio to the 0 h value. e Immunoblotting of REST in the cytoplasmic and nuclear fraction of PCCN in response to PrP106-126 stimulation. GAPDH and the nucleus-localized protein Lamin B demonstrate separation of the cytoplasmic and nuclear fractions. f REST level (normalized to GAPDH or Lamin B) in the nucleus and cytoplasm base on the immunoblots, shown as the ratio to the 48 h level. Data are represented as mean±SD of triplicate experiments.*P < 0.05, **P < 0.01 *versus* untreated cells (d) or cells treated with PrP106-126 for 48 h.

To determine whether neurons express REST protein and whether PrP106-126 alters REST protein expression, we examined REST protein expression by immunofluorescence and Western blot analysis. We have previously demonstrated that PrP106-126-FITC possesses similar cytotoxicity to PrP106-126 [26]. In this study, we treated PCCN with PrP106-126-FITC or untagged PrP106-126 fragments and directly observed the alteration of endogenous REST levels by immunofluorescence microscopy. As expected, after stimulation by PrP106-126-FITC or PrP106-126 for 24h, REST not only appeared to increase, but also had a tendency to translocate from the cytoplasm to nucleus (Figure 2a and 2b).

Then, we treated PCCN with 200μM PrP106-126 and determined REST protein concentrations at different time points (0h-48h) (Figure 2c). Consistent with the fluorescence results, the level of REST protein increased over time after incubation with PrP106-126 and peaked at 24 h. The level of REST was 1.6 to 1.9 fold higher than the control level at 6 to 24 h. Thereafter, the expression gradually declined to the control level by 48 h (Figure 2d), probably by polyubiquitylation and degradation of REST triggered by SCFβ^TrCP^ phosphorylation [29] [30] [31].

To further quantify the subcellular localization of REST in PCCN after incubation with PrP106-126, REST in cytoplasmic and nuclear fractions were analyzed by Western blotting (Figure 2e). REST expression in cytoplasmic and nuclear fractions was quantified using immunofluorescence and immunoblotting and GAPDH and Lamin B as the cytoplasmic and nuclear marker, respectively (Figure 2f). As expected, in agreement with the above results, upon stimulation with PrP106-126 for 24h, the amounts of REST in cytoplasm was decreased by 19.5% while REST in the nucleus increased (Figure 2b, 2e), compared with the untreated control group. The nucleus/cytoplasm REST ratio increased by approximately 6 and 10 folds at 24h and 48h, respectively, compared with the pretreatment ratio.

Taken together, we demonstrated that REST was activated and translocated from cytoplasm to nucleus in PCCN exposed to PrP106-126. This is the first report describing the induction and translocation of REST in primary neuronal cells in response to PrP106-126 stimulation.

### REST protects neurons from PrP106-126-induced synaptic damage and neurofibrillary degeneration

Although spongiform degeneration and neuronal death are cardinal features of prion diseases, the first detectable changes appear to be associated with neuronal dysfunction at the synapse [32] [33] [34]. REST is a gene-silencing factor and is widely expressed during embryogenesis in pluripotent stem cells and neural progenitors, where it acts *via* epigenetic remodeling to silence a large array of coding and noncoding neuronal genes important to synaptic function [14]. Meanwhile, emerging studies have shown the function of REST as a key mediator of presynaptic proteins in mature neurons under physiological [35] and pathological [36] [37] [38] [39] conditions. There have been only a few studies to examine postsynaptic proteins [40], especially in prion diseases [41]. Thus, we explored the potential relationship between neuronal REST and postsynaptic proteins in PrP106-126-stimulated PCCN.

**Figure 3:**
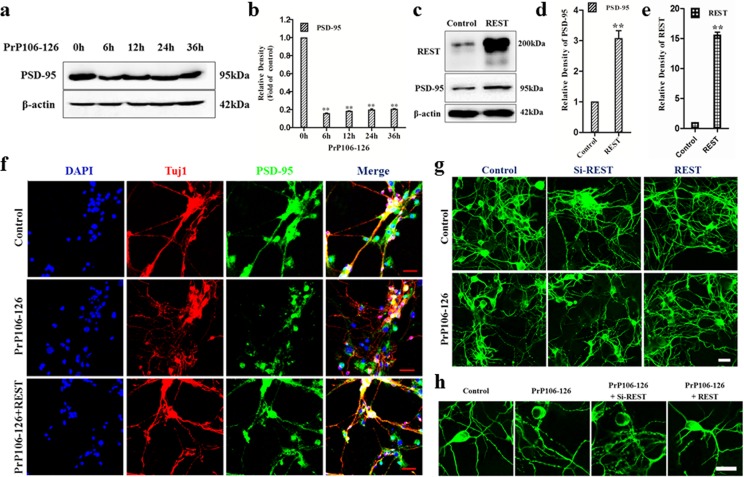
REST protects PCCN from PrP106-126 induced synaptic damage and neurofibrillary degeneration **a.**, **b.** Immunoblotting shows that PrP106-126 decreased the expression of the postsynaptic marker, PSD-95 in PCCN. PSD-95 level is expressed as ratio to the 0 h value. **c.**, **d.**, **e.** Immunoblotting analyses of PSD-95 and REST in PCCN transfected with HA-vector or REST-vector. REST and PSD-95 level are expressed as ratio of REST-transfected to the HA-vector transfected neurons. All data are represented as mean±SD of triplicate experiments and the values are normalized to the expression of β-actin. **P < 0.01 *versus* untreated cells (b) or cells transfected with HA-vector. **f.** Immunofluorescence microscopy shows that overexpression of REST restores the expression of PSD-95 in PCCN exposed to PrP106-126. Scale bars = 40μm. **g.** Overexpression of REST alleviates PrP106-126-induced neurofibrillary degeneration in PCCN. Knockdown of REST (Si-REST) exacerbates neuritic degeneration induced by PrP106-126. PCCN are incubated with PrP106-126 for 24 h. Neuritic processes are labelled with antibody Tuj1 (green). Scale bars = 20μm. **h.** Larger magnification of confocal photomicrographs in (g) to clearly show neuronal morphology. Scale bars = 10μm.

First, we examined the expression of PSD-95, a postsynaptic marker, in PCCN treated with 200 μM PrP106-126 for up to 36 h by immunoblotting to test if the neurotoxic peptide influences the level of PSD-95 (Figure 3a and 3b). Compared with the pre-treatment level, the expression of PSD-95 was markedly decreased after 6-36 h of PrP106-126 incubation (16% of the control value at 6 h).

As overexpression of REST protects cortical neurons from extensive neuritic degeneration after incubation with 5 mM oligomeric Aβ42 [16], we overexpressed REST by transfecting primary neurons with HA-tagged REST vector for 48 h and then examined the expressions of REST and PSD-95 (Figure 3c). As expected, compared with the empty HA vector-transfected control, REST overexpression significantly increased PSD-95 protein expression by 3 fold in PCCN when REST was overexpressed by about 15-fold (Figure 3d and 3e). To further investigate their relationship in pathological conditions, the PCCN were transfected with REST vectors for 48h, and then exposed to PrP106-126 (Figure 3f). The effect of REST overexpression on PSD-95 was further confirmed by immunofluorescence staining. Compared with the untreated control group, PSD-95-positive fluorescence intensity significantly decreased in the PrP106-126-treated group, associated with marked neurite degeneration, as indicated by extensively beaded, dystrophic neurites and abnormal microtubule cytoskeleton (Figure 3f). Additionally, the expression of PSD-95 was relatively rescued by the transduction of REST, showing similar distribution to the negative control (Figure 3f). These results suggest that REST plays an important role in maintaining postsynaptic integrity both in physiological and pathological conditions.

**Figure 4:**
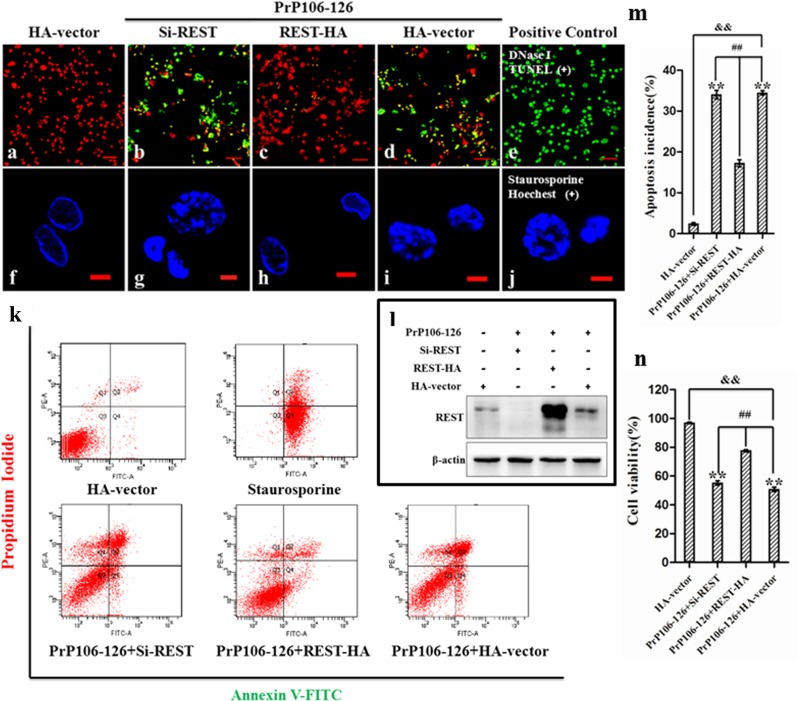
REST is neuroprotective in response to PrP106-126-induced neuronal death PCCN cells are transfected with a control vector (HA-vector), REST siRNA or REST vector to knockdown or overexpress REST, respectively, and then exposed to PrP106-126 for 24 h. **a.**-**e.** TUNEL assay shows increased apoptosis in cells with REST knockdown and decreased apoptosis with REST overexpression. Apoptotic cells are labelled with the in situ cell death kit, POD and counterstained with propidium iodide (red). DNase I = positive control. Scale bar = 50μm. **f.**-**j.** Hoechst 33258 staining shows that REST overexpression rescued cells from nuclear fragmentation and condensation in response to PrP106-126. Staurosporine = positive control. Scale bar = 5μm. **k.** Flow cytometry analysis shows REST overexpression reduces apoptosis of both early and late stages induced by the prion peptide. Staurosporine = positive control. **l.** Immunoblotting analysis of REST in PCCN. **m.**, **n.** Inhibition of cell apoptosis (m) and increased cell survival (n) of PCCN by REST overexpression and treated with PrP106-126 were confirmed by flow cytometry. Data are mean ± S.D. of three independent experiments. **P < 0.01 and ^&&^ *P* < 0.01 both *versus* cells treated without PrP106-126; ^##^ *P* < 0.01 *versus* cells transfected with REST-vector.

Synaptic dysfunction is followed by dendritic loss, which correlates with neurofibrillary degeneration in the early stages of neuropathological change in prion diseases [42] [5]. Following the above observations of the effects of REST on neurons, we further evaluated changes in neuronal morphology after REST overexpression or silencing. SiRNA-mediated REST knockdown was confirmed by western blot analysis (Figure 4l). Upon treatment with PrP106-126, as expected, REST-knockdown neurons showed markedly increased collapse of the cytoskeleton and neurofibrillary degeneration. REST overexpression rescued the PrP106-126-treated PCCN from the disruption of microtubule cytoskeleton (Figure 3g).

At higher magnification we clearly observed some of the key cytopathological lesions of different treated groups (Figure 3h). As expected, the most serious neurofibrillary degeneration and dystrophic neurites occurred in the REST deficient neurons exposed to PrP106-126, as shown by diffusively and slightly beaded neurites. Taken together, these results support the critical role of REST in regulating postsynaptic integrity and protecting primary neurons from PrP106-126-induced neurofibrillary degeneration.

### REST is neuroprotective and regulates expression of proteins involved in neuronal cell death induced by PrP106-126

Perturbation of REST expression during embryogenesis causes cellular apoptosis, aberrant differentiation and patterning, and lethality [15]. REST represses genes involved in cell death and acts as a neuroprotective factor in AD [16], mediates intrinsic homeostasis, and protects neuronal networks from hyper excitability [43] [28]. We therefore explored the role of REST in PrP106-126-induced neuronal death by detecting the hallmarks of apoptosis.

**Figure 5:**
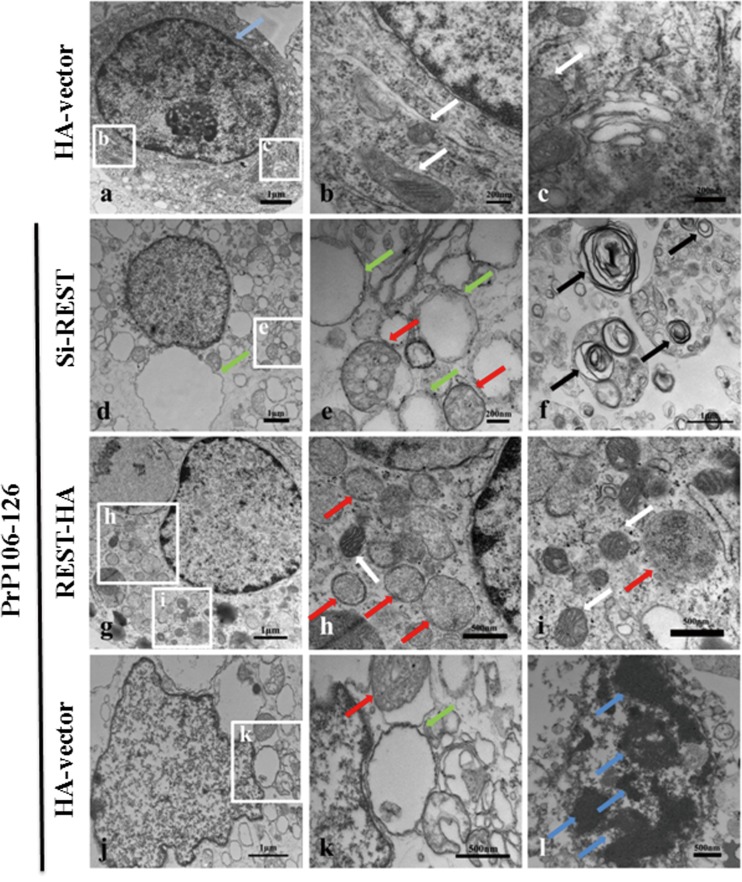
REST protects PCCN cells from PrP106-126 induced morphological changes PCCN cells are transfected with the control vector (HA-vector) without exposure to PrP106-126 **a.**-**c.**, or transfected with the control vector **j.**-**l.**, REST siRNA **d.**-**f.** or REST vector **g.**-**i.** and then exposed to PrP106-126 for 24 h as described in Material and Methods. Transmission electron microscopy shows normal morphology of nucleus (light blue arrows) (a) and mitochondria (white arrows) (b, c) in the negative control, and various sizes of vacuoles or swollwn and disorganized smooth endoplasmic reticulum (green arrows) as well as swollen and vacuolization of mitochondria (red arrows) in siRNA REST (d, e) or HA-vector (k) transfected cells. Black arrows or blue arrows indicate the presence of myelin figures (f) or nuclear fragmentation (l). PCCN transfected with REST and then exposed to PrP106-126 show normal morphology of organelles and barely visible vacuolization with only slightly swollen mitochondria (h, i).

First, we overexpressed or blocked REST as described above and then treated neuronal cells with 200 μM PrP106-126. HA vectors were transfected as a negative control and the treatment of staurosporine (Figure 4e and 4j) (500 nm for 24h) [44] as a positive control of neuronal death. REST protein levels were determined by Western blotting (Figure 4l). We measured neuronal apoptosis by TUNEL assay and Hoechst staining. After stimulation by PrP106-126, the Si-REST and HA-vector groups both showed obvious apoptosis as shown by intense TUNEL-positive (green) staining, whereas the REST overexpression group had only minor apoptosis as shown by weak and scattered green fluorescence intensity (Figure 4a-4d). Hoechst staining was performed to confirm the presence of apoptotic body formation, and showed nuclear changes including nuclear shrinkage and chromatin condensation (Figure 4f-4i), consistent with the TEM findings (Figures 1 & 5).

Flow cytometric analysis using the Annexin V-FITC/PI apoptosis detection kit provided quantitative data on PrP106-126-induced apoptosis of PCCN. Compared with the siRNA-transfected (55.2% viability) and HA-vector (50.8%) groups, REST-overexpression significantly increased cell viability (77.5%) (Figure 4k and 4n). Including the early and late stage of apoptosis, apoptosis of PrP106-126-treated PCCN was significantly decreased after transfection with REST (17.2%), relative to the si-REST (34.0%) and HA-vector (34.5%) groups (Figure 4k and 4m).

The neuropathological damage of PCCN in different groups was also assessed by TEM. Upon treatment with PrP106-126, the Si-REST (Figure 5d-5f) and HA-vector (Figure 5j-5l) groups had similar ultrastructural pathology as described above. Compared with the negative control (Figure 5a-5c), mitochondria of the REST siRNA-transfected and prion peptide-treated neurons decreased in number and had severely swollen vesicles or vacuoles and the absence of or fractured cristae, and endoplasmic reticulum displayed varied sizes and shapes of vacuoles. Organelles of neurons transfected with REST remain largely intact with very minor vacuolization and particularly, mitochondria were intact, with occasional loose or swollen cristae (Figure 5g-5i). Together, these observations indicated that REST protected PCCN ultrastructure against PrP106-126-induced neuropathological damage.

**Figure 6:**
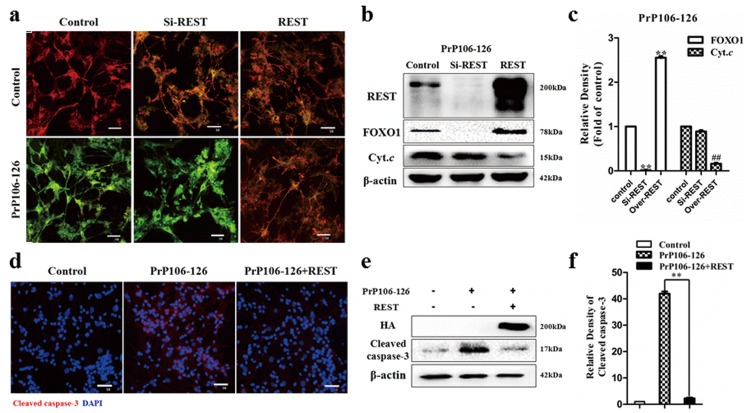
**a.** REST protects neurons from mitochondrial dysfunction induced by PrP106-126. Mitochondrial transmembrane potential (MTP) is measured using JC-1 as the fluorescent marker. The JC-1-aggregate form, indicating normal MTP, appears red and the monomeric form, indicating low MTP (i.e., disrupted mitochondrial membrane), is green by confocal microscopy. Scale bar = 20 μm. **b.**-**f.** REST regulates expression of cell death-related proteins induced by PrP106-126. b and c REST, FOXO1 and cytochrome *c* (*Cyt.c*) proteins are examined by immunoblotting and immunoblotting density was normalized to β-actin and expressed as ratio to the HA-vector and PrP106-126 treated control group. Data are presented as mean±SD of triplicate experiments. **P < 0.01 and ^##^ *P* < 0.01 *versus* the control group. d-f Immunofluoresence and immunoblotting show that REST inhibited the activation of caspase 3 (cleaved form). d. Nuclei (blue) are stained with DAPI. Scale bar = 50 μm. e Overexpressed REST is confirmed by anti-HA antibody. f Cleaved caspase 3 is normalized to β-actin expressed and expressed as ratio to the untreated control group. Data are presented as mean±SD of triplicate experiments. **P < 0.01 *versus* the REST-vector and PrP106-126 treated group.

We further explored the potential downstream mechanism of REST acting as a neuroprotective factor. Since mitochondria are critical regulators of cell survival and death in neurodegeneration [45], we assessed mitochondrial function by the JC-1 Mitochondrial Transmembrane Potential (MTP) Assay (Figure 6a). In concert with previous findings, after stimulation by PrP106-126, the Si-REST and HA-vector groups displayed increased green fluorescence (JC-1 monomer form) and decreased red fluorescence (JC-1 aggregates form), indicating low MTP values and thus mitochondrial dysfunction compared with the negative control cells. The overexpression of wild type REST reduced PrP106-126-induced JC-1 monomers and increased JC-1 aggregates, indicating normal or improved mitochondrial function. Under the same condition, we examined two cell death-associated proteins by western blotting (Figure 6b and 6c). The results demonstrated that the overexpression of REST greatly repressed the release of cytochrome *c* (16.2% of the negative control), which acts downstream in the mitochondrial apoptotic pathway, and enhanced the expression of the anti-apoptotic protein, transcription factor FOXO1 (by 2.6 fold relative to the negative control), which mediates oxidative stress resistance [46] [47] and is protected by REST in AD [16]. REST knockdown by siRNA transduction almost completely abolished FOXO1 expression. Activation of caspase-3 is a necessary but insufficient event in the execution of apoptotic cell death and also a feature of many chronic neurodegenerative diseases [48]. We examined whether overexpression of REST inhibits the activation of caspase-3 by immunofluorescence (Figure 6d) and immunoblotting (Figure 6e and 6f). Consistent with our hypothesis, REST overexpression by REST-vector-transfection significantly reduced caspase-3 activation induced by the prion peptide. HA antibody analysis confirmed the overexpression of REST. Taken together, we assumed that REST acts as a neuroprotective regulator in PrP106-126-induced neuronal death by repressing pro-apoptotic proteins and/or promoting the expression of anti-apoptotic proteins.

### Regulation of REST by the Wnt-β-catenin signaling pathway

We further explored the possible signaling pathway of REST as a neuroprotective factor in PrP106-126-induced neuronal death. The canonical Wnt-β-catenin pathway had been shown to regulate the expression of REST in chick spinal cord [49] or AD [16] in physiological or pathological conditions. Wnt ligands activate the pathway by binding to the low-density lipoprotein receptor-related protein 6 (LRP6) together with Frizzled receptors and transduce signals through the stabilization of β-catenin. The stabilized β-catenin in turn translocates to the nucleus, where it activates Wnt target genes [50]. The loss of β-catenin signaling increases neuronal vulnerability to apoptosis induced by amyloid-β protein [51]. Conversely, increased activity of glycogen synthase kinase 3 (GSK3β), which inhibits the LRP6-mediated Wnt-β-catenin signaling, is believed to account for several pathological hallmarks of prion peptide-induced cell death [52]and AD [53, 54]. We examined β-catenin and GSK3β as the indicator of activation and inhibition, respectively, of the Wnt-β-catenin signaling pathway.

**Figure 7:**
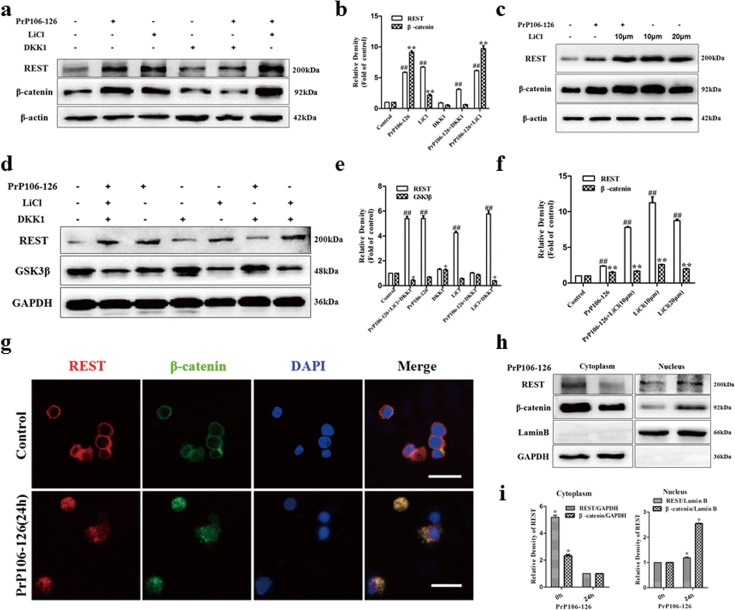
Regulation of REST by the Wnt-β-catenin signaling pathway **a.** and **b.** Immunoblotting shows that the induction of REST and β-catenin by PrP106-126 or the activator of Wnt signaling, lithium chloride (LiCl), is partially suppressed by the Wnt antagonist Dickkopf (DKK1). **c.** and **f.** Immunoblotting reveals LiCl increased the levels of REST and β-catenin in a dose-dependent manner. REST and β-catenin are normalized to β-actin and expressed as ratio to the control group. Data are presented as mean±SD of triplicate experiments. ^##^ *P* < 0.01 and **P < 0.01 *versus* the control group, respectively. **d.** and **e.** Immunoblotting shows that REST is negatively correlated with GSK3β in the Wnt signaling. REST and GSK3β are normalized to β-actin and expressed as ratio to the control group. Data are presented as mean±SD of triplicate experiments. ^##^ *P* < 0.01 and *P < 0.05 *versus* the control group. **g.** Translocation and colocalization of REST and β-catenin in the nucleus after exposure to PrP106-126. The PCCN are double-labelled for REST (red) and β-catenin (green). Nuclei (blue) are stained with DAPI. Scale bars = 10μm. **h.** Immunoblotting confirms the translocation and colocalization of REST and β-catenin as indicated. Cytoplasmic and nuclear fractions are separately collected and the fractions are immunoblotted for REST. GAPDH and the nucleus-localized protein Lamin B demonstrate separation of the cytoplasmic and nuclear fractions. **i.** Immunoblotting density of REST and β-catenin in cytoplasm or nucleus are normalized to GAPDH or Lamin B, respectively, and expresse as ratio to the cytoplasm fraction at 24 h and the nuclear fraction at 0 h. Data are presented as mean±SD of triplicate experiments. *P < 0.05 *versus* cells at 24h for cytoplasm fraction and the nuclear fraction at 0 h for the nuclear fraction.

PCCN was incubated with PrP106-126 (300 μm) for 24h. β-Catenin in parallel with REST increased significantly in the prion peptide-treated cells, compared with the negative control (Figure 7a, 7b). The GSK3-β inhibitor, lithium chloride (LiCl) activated Wnt signaling induced-β-catenin as well as REST (Figure 7a-7c and 7f). Treatment with both PrP106-126 and LiCl enhanced the increase of β-catenin and REST compared with the negative control. By contrast, induction of REST and β-catenin was partially inhibited by the Wnt signalling antagonist, Dickkopf (DKK1) (Figure 7a and 7b). In concert with our hypothesis, immunoblotting showed a significant inverse relationship between REST and GSK3-β after the neruronal cells were incubated with PrP106-126, LiCl or DKK1 as indicated (Figure 7d and 7e). Furthermore, immunofluorescence (Figure 7g) and immunoblotting data (Figure 7g-7i) demonstrated that REST translocated from cytoplasm to nucleus in PCCN after stimulated by PrP106-126 and colocalized with β-catenin. These results indicate that Wnt-β-catenin signaling may contribute to the induction of REST in PrP106-126-induced neuronal death.

### LRP6 is critical for the activation of REST and protects neurons from PrP106-126-induced synaptic and neurofibrillary degeneration

**Figure 8:**
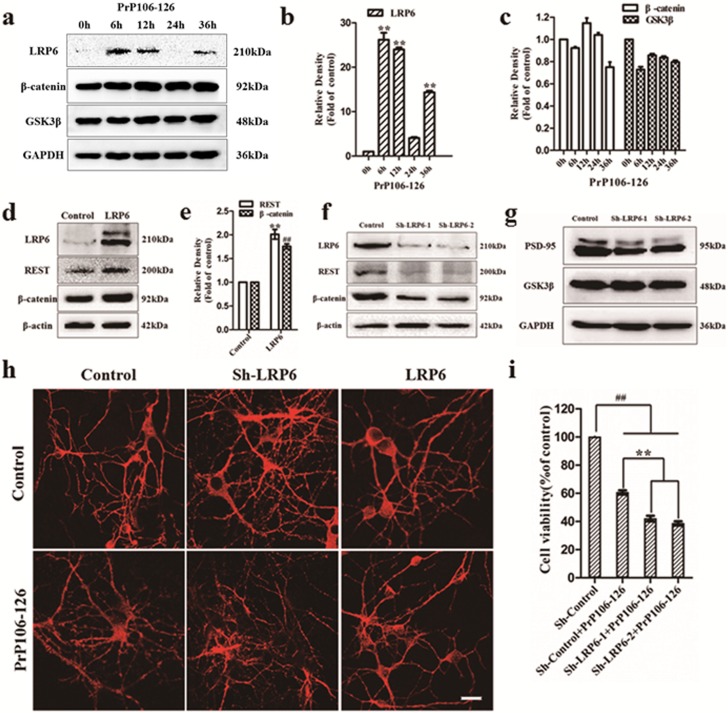
**a.-c.** LRP6 expression in PCCN exposed to PrP106-126. Immunoblotting density of LRP6, β-catenin and GSK3β was normalized to the expression of GAPDH and expressed as ratio to the untreated group. Data are presented as mean±SD of triplicate experiments. **P < 0.01 *versus* the untreated control group. **d.** and **e.** Overexpression of LRP6 markedly induces the expression of REST and β-catenin. Immunoblotting density of LRP6, REST and β-catenin were normalized to β-actin and expressed as ratio to the HA- vector control group. Data are presented as mean±SD of triplicate experiments. **P < 0.01 *versus* the control group. **f.** LRP6 knockdown (sh-LRP6-1 and sh-LRP6-2) inhibits the expression of REST and β-catenin. The expression of LRP6, REST and β-catenin were examined by immunoblotting in the control and knockdown of LRP6 group. **g.** LRP6 knockdown inhibits the level of PSD-95. The expression of LRP6, PSD-95 and GSK3β were examined by immunoblotting in the control and knockdown of LRP6 group. **h.** LRP6 protects against PrP106-126 induced neurofibrillary degeneration. Knockdown of LRP6 also show extensive neuritic degeneration relative to control after incubation with PrP106-126 for 24 h, which is prevented by overexpression of LRP6. Neuritic processes are labelled with antibody Tuj1, red. Scale bars = 20μm. **i.** Knockdown of LRP6 significantly aggravated PrP106-126 induced neuronal death. Quantification of the cell viability incidence tested by flow cytometry. All the above data were presented as the mean ± S.D. of three independent experiments. **P < 0.01 *versus* cells transfected by sh-control vector and treated with PrP106-126; ^##^ *P* < 0.01 *versus* PCCN transfected by sh-control vector only group.

Dickkopf-1 (DKK-1) is an extracellular secreted inhibitor of the canonical Wnt pathway by binding to the co-receptor LRP6 [19] [18]. Moreover, Bu and colleagues reported that LRP6-mediated Wnt signaling in neurons is critical for synaptic integrity and neuronal viability [23]. Therefore, we further investigated the influence of PrP106-126 on the canonical Wnt pathway and the relationship between LRP6 and REST. First, PCCNs were treated with 200 μM PrP106-126 and the total protein were harvested at different time points (0h-36h) for the determination of the expression of LRP6, β-catenin and GSK3β (Figure 8a, 8b). LRP6 showed a transient increase after incubation for 6h to 12h and then recovered nearly to the pre-treatment level at 24 h. However, with prolonged stimulation by the prion peptide, LRP6 protein increased again at 36 h. β-catenin and GSK3β had no significant changes (Figure 8c). Then, we explored the expression of REST and β-catenin after LRP6 overexpression or short hairpin RNA-mediated knockdown of LRP6. As expected, overexpression of LRP6 markedly increased the levels of REST and β-catenin by 2 and 1.8 fold relative to the HA-vector transfected negative control (Figure 8d, 8e and S2 a). Correspondingly, upon LRP6 knockdown with either of the two distinct short hairpin RNAs (shRNAs) (Sh-LRP6-1 and Sh-LRP6-2), the levels of both REST and β-catenin significantly decreased to approximately 20% and 30%, respectively, of the negative control transduced with an empty vector (Figure 8f, 8g; S2), indicating the suppression of Wnt signaling and associated functions.

We also evaluated changes in neuronal morphology following LRP6 shRNA transfection by immunofluorescence (Figure 8h). Prolonged LRP6 knockdown induced pronounced neurofibrillary fracture, showing extensive neurite fragmentation. PrP106-126 treatment exacerbated the neurofibrillary degeneration and loss in the PCCN transfected with the LRP6 shRNA vector. As LRP6-mediated Wnt signaling plays an important role in maintaining postsynaptic integrity and neuronal viability in AD [23], we further examined the expression of PSD-95 and GSK3β in the LRP6-konckdown PCCN. Consistent with our expectation, PSD-95 decreased to 42-60% of the negative control value (Figure 8i and 8j; S2 c), but GSK3β was unaffected. Additionally, flow cytometric analysis using the Annexin V-FITC/PI apoptosis detection kit showed that LRP6 knockdown significantly aggravated PrP106-126-induced neuronal death, with neuronal viability approximately 40%, compared with 60% in the untreated, LRP6 sh-RNA-transfected group (Figure 8i). Taken together, these results support the hypothesis that LRP6-mediated Wnt-β-catenin signaling increases the expression of REST and maintains postsynaptic integrity and neuronal viability in response to PrP106-126 neurotoxicity.

## DISCUSSION

### Activation and translocation of REST is a universal feature in response to stressors

We demonstrated that REST is inducible by PrP106-126, which has similar neurotoxicity and pathogenic properties to PrP^Sc^ *in vitro* and in some animal models [55], and translocates from the cytoplasm to the nucleus in PCCN upon exposure to the prion peptide, although REST expression decreased after peaking at 24 h. The decline was probably due to degradation by proteasomes [56] [57] [58, 59], eventually failing to function as a neuroprotective factor with prolonged exposure to PrP106-126. Previously, REST was reported to be inducible by various neurotoxic stimuli, nutrients or neuronal activity including hydrogen peroxide, oligomeric Aβ42 [16], the glycolytic inhibitor 2-deoxy-D-glucose (2DG) [43] and kainic acid (KA)-induced seizures [60]. Activation and translocation of REST is a universal feature in response to stressors.

Nucleocytoplasmic transport is considered an important event for REST as a transcriptional repressor to regulate neuronal gene expression. The phenomenon of protein shuttling between the nucleus and cytoplasm is also an important mechanism in regulating apoptosis and maintaining the basal activities of transcription factors by signaling molecules [61] [62] [63]. Our findings and other published studies suggest that nuclear REST is a key factor of neuroprotection in prion diseases, AD and other neurodegenerative diseases. The dynamics of REST levels in neurons and non-neurocytes could be due to miscellaneous regulatory mechanisms. [64] [65, 66]. The cytoplasm to nucleus transport mechanism and association of REST with specific cellular partners in response to PrP106-126 in neurons should be further elucidated.

### REST maintains cellular integrity in PrP106-126-induced morphological damage

Previous studies have demonstrated that by maintaining neural progenitor cells in a self-renewing state, REST maintains plasticity both in embryonic development and adult neurogenesis [67] [68] [69] in physiological conditions. On the other hand, the hallmark neuropathological changes in prion diseases begin with synaptic alterations [4]. The successive dysfunctions followed by spongiform degeneration consists of diffuse or focally clustered, small, round or oval vacuoles in the neuropil of the deep cortical layers, cerebellar cortex or subcortical gray matter [70, 71]. PrP106-126 prion peptide is present in several longer peptides isolated from cerebral amyloid plaques of patients suffering from Gerstmann-Sträussler-Scheinker disease (GSS) [72], and is cytotoxic *in vivo* after injection into mouse retina [55]. As a convenient and neurotoxic model for *in vitro* studies of prion-induced neuropathological changes [9, 10, 26], PrP106-126 was demonstrated to have a similar cytopathic effect to PrP^Sc^ on PCCN by TEM in our study. REST maintained the neuronal integrity by stabilizing the expression of the postsynaptic protein, PSD-95. Moreover, REST overexpression protected neurons from neurofibrillary degeneration through restoring some structural integrity of subcellular organelles and reducing vacuolization. REST provides a hub that coordinately regulates multiple tiers of neuronal development [14, 38] in physiological as well as in pathological conditions by complex and multifactorial processes.

### REST is a neuroprotective regulator in PrP106-126-induced neuronal death

Perturbation of REST expression or function results in early embryonic lethality and ectopic expression of neuronal genes in non-neuronal tissues [15, 67]. Recently, Tao Lu and colleagues have shown that REST protein protects ageing neurons from death by repressing genes that promote cell death and AD pathology and inducing the expression of stress response genes [16]. By overexpression or silencing of REST in PCCN exposed to PrP106-126, we demonstrated that REST works as a neuroprotective regulator and contributes to neuronal viability by stabilizing the level of pro-survival protein FOXO1 and the permeability of the mitochondrial outer membrane, and inhibiting the release of cytochrome *c* from mitochondria to cytosol and its downstream activation of Capase3. Mitochondria are critical regulators of cell survival and death in neurodegeneration [45] and previous data demonstrated that REST-mediated transcriptional repression confers resistance to some pro-apoptotic genes in AD, including the mitochondrial permeability transition pore ANT1 (also known as SLC25A4) and cytochrome *c* [16]. Our results are consistent with these findings. We propose that the transiently elevated protein levels of REST induced by PrP106-126 initially maintain homeostasis and plasticity in neurons. REST-mediated transcriptional repression should confer resistance to pro-apoptotic genes, but REST then is degraded by proteasomes and fail to function as a neuroprotective factor with continued stimulation by stressors [56, 58].

### REST plays a novel and crucial role in LRP6-mediated Wnt-β-catenin signaling in prion diseases

In the Wnt-β-catenin pathway, there are several genes that influence REST transcription associated with β-catenin [49]. We confirmed that the activator of Wnt signaling, lithium chloride (LiCl), increased the expression of REST in parallel with β-catenin in a dose-dependent manner in PCCN. Moreover, REST is correlated with β-catenin in expression and co-localizes with β-catenin in the nucleus after stimulation by PrP106-126. In contrast, the Wnt signaling antagonist Dickkopf-related protein 1 (DKK-1) inhibited the expression of REST. Lu et al. reported that the increased expression of the REST protein is at least partially dependent on the Wnt-signaling pathway in the ageing brains of healthy people [16]. Meanwhile, the Wnt signaling pathway has long been associated with the modulation of neurogenesis, dendritic morphogenesis, and synaptic function [17-19]. In the canonical Wnt pathway, Wnt ligands bind to its receptor, Frizzled (Fz) and the associated co-receptor LRP5/6 to activate disheveled, which inhibits the GSK-3β kinase and prevents the phosphorylation and subsequent degradation of β-catenin. By contrast, DKK binds to and inhibits LRP5/6, blocking the canonical Wnt pathway [73]. Moreover, LRP6-mediated Wnt signaling has recently been reported to play a critical role in AD. LRP6 deficiency contributes to synaptic abnormalities and amyloid pathology. We therefore further explored the relationship between REST and LRP6 in the Wnt-β-catenin signaling. Excitingly, this study provides the first observation that LRP6 evidently regulates the expression of REST in addition to β-catenin. Consistent with the previous data, LRP6 is also critical for maintaining PSD-95 expression, cellular morphological integrity and neuronal viability in PrP106-126-induced cytopathology. Several studies have also shown that β-catenin acts as an important mediator of dendritic development and plays an important role during cellular morphogenesis [74]. We assume that at the beginning of exposure to PrP106-126, the LRP6 receptor is stimulated by stress responses and in turn activates Wnt-β-catenin signaling, resulting in the transiently elevated protein levels of REST and β-catenin followed by translocating from the cytoplasm to the nucleus. Taken together, in the PrP106-126-induced neuropathology in PCCN, both REST and LRP6-Wnt-β-catenin signaling are critical for maintaining cellular integrity and protecting neurons from neuronal death. They work cooperatively to maintain neuronal homeostasis. However, activation of LRP6-Wnt-β-catenin signalling is also implicated in the development of various cancers [75]. Thus, this approach would probably require careful targeting of LRP6-Wnt-β-catenin /REST activation without triggering other diseases. A deeper understanding of the molecular mechanisms that govern REST activation in neurodegeneration will be crucial for such a therapeutic strategy to be successful.

## CONCLUSIONS

Our present data indicates that REST acts as a novel and critical neuroprotective factor, which is partially regulated by the LRP6-Wnt-β-catenin signaling (S3). Our study demonstrates that this signaling pathway may be important for the basic regulatory mechanism of neuron survival in prion diseases and associated neurodegenerative diseases.

## MATERIALS AND METHODS

### Animal ethics statement

All of the animal experiments were conducted in accordance with the guidelines of Beijing Municipality on the Review of Welfare and Ethics of Laboratory Animals and approved by the Beijing Municipality Administration Office of Laboratory Animals (BAOLA).

### Reagents

The rabbit polyclonal anti-REST antibody (07-579) (1:500) was purchased from Millipore. The goat polyclonal anti-NRSFantibody (P-18) (sc-15118), the mouse monoclonal anti-cytochrome c antibody ((A-8) sc-13156) (1:200), the mouse monoclonal anti-Bcl-1 antibody (C-2)(sc-7382)(1:200) were purchased from Santa Cruz Biotechnology (Santa Cruz, CA, USA). The rabbit polyclonal anti-REST antibody (22242-1-AP)(1:200), the rabbit polyclonal anti-PSD-95 antibody (20665-1-AP) (1:200), the rabbit polyclonal anti-beta-Catenin antibody (51067-2-AP) (1:200), the rabbit polyclonal anti-GSK3β antibody (22104-1-AP) (1:200), the mouse monoclonal anti-GAPDH antibody (60004-1-lg) (1:1000), the rabbit polyclonal anti-Cleaved Caspase 3,p17-specific antibody(25546-1-AP) (1:200), the rabbit polyclonal anti-Lamin B1 antibody (12987-1-AP) (1:500) were purchased from Proteintech Biotechnology (Chicago, USA). The rabbit monoclonal anti-FoxO1 antibody (C29H4)(#2880) (1:500), and the rabbit polyclonal anti-LRP6 antibody (C5C7)(#2560) (1:200) were purchased from Cell Signaling Technology(Danvers, Massachusetts, USA). The mouse monoclonal anti-HA-Tag antibody (Cat No.: AP0005M) (1:1000), the rabbit polyclonal anti-rat Bax(S163) antibody (Cat No.: BS1725) (1:200), the rabbit polyclonal anti-rat β-actin antibody (Cat No.: AP0060) (1:1000), the goat anti-rabbit IgG(H&L)-HRP secondary antibody(Cat No.:BS13278) (1:5000) were purchased from Bioworld Technology (Nanjing, China). The goat anti-mouse IgG(H&L)-HRP secondary antibody (1:100), TRITC-conjugated AffiniPure Rabbit Anti- Goat IgG(H+L)(ZF-0317) secondary antibody (1:100), Alexa Fluor 594-Conjugated AffiniPure Goat Anti-Rabbit IgG(H+L) (ZF-0516) (1:100), Peroxidase-Conjugated Affinipure goat anti-rabbit IgG(H+L) (ZB-2301) (1:5000)and rabbit anti-goat IgG(H+L)(ZB-2306) (1:5000)were purchased from Beijing ZSGB Biotechnology (Beijing, China); Donkey anti-rabbit IgG/FITC(bs-0295D-FITC) (1:100) and Donkey Anti-Goat IgG/Cy3 (bs-0294D-Cy3) (1:100) secondary antibodies were purchased from Beijing Biosynthesis Biotechnology(Beijing, China); Recombinant Rat DKK-1 (4010-DK-010) was purchased from R&D systems (Minneapolis, MN, USA), the Neuronal Class III β-Tubulin (Tuj1) antibody(AT809) (1:250), DAPI dihydrochloride and propidium iodide (PI) were purchased from Beyotime Biotechnology (Wuhan, Hubei, China). Reagents and apparatus used in immunoblotting assays were purchased from Bio-Rad (Richmond, CA, USA).

### Prion protein peptide

PrP peptides PrP106-126 (sequences KTNMKHMAGAAAAGAVVGGLG), were synthesized by Sangon Bio-Tech (Beijing, China). The purity of prion peptides was > 95 % according to the data from the synthesizer. FITC-labeled PrP106-126 peptides were purchased from FANBO BIOCHEMICALS (Beijing, China). The peptides were dissolved in 0.1 mol/l PBS to a concentration of 1 mmol/l, and left to aggregate at 37°C for 24 hours before each experiment. Experiments were conducted with final peptide concentrations of 200μM or 300μM.

### Primary cultured cortical neurons (PCCN)

Dissociated cerebral cortex neuronal cultures were prepared from postnatal 1-day-old Sprague-Dawley rat, according to the previously described procedure [26, 76]. Briefly, cells were gently dissociated after digestion with papain (Invitrogen, Carlsbad, CA, USA). The dissociated cells were plated at a final density of 5×10^5^cells/cm^2^ on polyethyleneimine (Sigma-Aldrich, St. Louis, MO, USA)-coated plates and cultured in DMEM F12 (Hyclone, Logan, UT, USA), supplemented with 2% B27 (Invitrogen), and 0.5% Penicillin-Streptomycin (Gibco, Grand Island, NY, USA). Two days later, 10μM cytarabine (Sigma) were added to repress the growth of glial cells.

### Plasmids and transfection

The pCMV-HA-Rest vector of Full-length REST (Cat No. PPL50007-2a), the pCMV-C-HA-LRP vector of Full-length LRP6 (Cat No. PPL00515-2a) were obtained from Public Protein/Plasmid Library (Nanjing, China). The pGPH1/GFP/Neo-LRP6-Rat shRNA-1 vector and shRNA-2 vector were obtained from GenePharma (Suzhou, China). For transfection [76] [26], cultured primary neurons were washed with Opti-MEM (Invitrogen, Carlsbad, CA, USA) and then transfected with the appropriate plasmids using the Lipofectamine 2000 reagent (Invitrogen) in Opti-MEM without serum according to the manufacturer's instructions. The culture medium was replaced by the primary cell culture medium 5 h after transfection. Forty-eight hours after transfection, the cells were observed using an upright fluorescence confocal microscopy (Olympus, Tokyo, Japan) or subjected to immunoblot analyses.

### Small interfering RNA transfection

Small interfering siRNA targeting REST (Cat. No. 1027416, Qiagen, Valencia, CA, USA) was used for silencing REST expression. Primary neurons were plated at 1×105 cells/well in a 12-well plate for 24 h before transfection with siRNA in accordance with the manufacturer's instructions. Briefly, on the day of transfection, 75 ng siRNA was diluted in 100 μl culture medium without serum. A volume of 5.5 μl of the transfection reagent (HiPerfect Transfection Reagent, Qiagen, Valencia, CA, USA) was added to the diluted siRNA and mixed by vortex. The samples were then incubated for 5 to 10 min at room temperature to allow the formation of transfection complexes before adding the complexes into the cell culture. REST expression was determined by western blotting.

### Immunofluorescence

For protein localization analysis, primary neuronal cells grown on cover slips were washed twice with PBS, fixed by Immunolo Staining Fix Solution (P0098), blocked 1h at room temperature by Immunol Staining Blocking Buffer (P0102) and then incubated overnight at 4°C with the appropriate primary and secondary antibodies. The nuclei were stained with DAPI. Primary neuronal cells were labelled using anti-Tuj1 (neuronal class III tubulin) antibody (Beyotime, Wuhan, Hubei, China) and fluorescein isothiocyanate (FITC)-conjugated donkey anti-mouse antibodies as the primary and secondary antibodies, respectively.

To visualize the localization of PrP106-126 in primary neuronal cells, we treated the cells with the FITC-labeled PrP106-126 in culture medium for 24 h before observed, then treated them as above. The treated cells were observed under an upright fluorescence confocal microscope. All reagents for fixation, wash and blocking were purchased from Beyotime Biotechnology(Wuhan, Hubei, China).

### Protein extraction and western blotting

Protein extraction and Western blotting was performed as described previously [26, 76].

### Terminal deoxynucleotidyl transferase dUTP nick end labeling (TUNEL) assay

TUNEL analysis was performed to determine cellular apoptosis using an in situ cell death detection kit, POD (Roche, Basel, Switzerland,) following the manufacturer's instructions. Primary neurons were grown on cover slips in poly-D-lysine-coated 12-well Lab-Tek^®^ culture dishes at a density of 5×10^5^cells/well. Cells were counterstained with propidium iodide (PI) for nuclei. The TUNEL Positive control kit (C1082) was purchased from Beyotime Biotechnology (Wuhan, Hubei, China). All the slides were visualized using an upright fluorescence microscope (Olympus Fluoview, Japan).

### Morphological observation of apoptotic cells by Hoechst 33258 staining

Morphological assessment of apoptotic cells was performed using the Hoechst 33258 staining method. Osteoblasts were prepared at a density of 5,000 cells per well in a 24-well plate. Apoptotic cells were detected using the Hoechst 33258 staining (Beyotime, Haimen, China) and trypan blue (Beyotime, Haimen, China) exclusion assay. The cells were fixed with 4 % paraformaldehyde for 10 min, washed with PBS for three times and then stained with 2 μg/ml Hoechst 33258 for 5 min. Morphologic changes in apoptotic nuclei were evaluated under a fluorescence microscope (excitation wavelength 350 nm, emission filter 460 nm) (Olympus Fluoview, Japan).

### Measurement of apoptosis by flow cytometry

Apoptotic cell counting was detected by using the Annexin V-FITC/PI apoptosis detection kit (KGA105-KGA108) (KeyGEN BioTECH, Nanjing, China). Apoptotic cells, including those staining positive for Annexin V-FITC, negative for PI and double positive cells were counted.

### Transmission electron microscopy (TEM)

Primary neurons were treated and then washed twice in PBS, trypsinized, fixed in ice-cold 5 % glutaraldehyde in 0.1 M sodium cacodylate buffer (pH 7.4) at 4°C for 15 min, and then centrifuged. Cell pellets were fixed for 4 h. After a complete rinse with sodium cacodylate buffer, the cell pellet was further fixed in 1 % OsO4 in 0.1 M sodium cacodylate buffer on ice for 1 h and dehydrated with acetone. The cell pellet was embedded in EM-bed 812 resin and polymerized at 60°C for 48 h. Ultrathin sections (70 nm) were obtained on a Leica Ultracut UCT ultramicrotome (Vienna, Austria) and counterstained with uranyl acetate and lead citrate before observation under a fluorescence microscope (Olympus Fluoview, Japan).

### Mitochondrial transmembrane potential (MTP) assay

Mitochondrial transmembrane potential was evaluated using the cationic fluorescent indicator JC-1 (Molecular Probes, Eugene, OR, USA), which aggregates in intact mitochondria (red fluorescence) with normal MTP, but remains in the monomeric form in the cytoplasm (green fluorescence) of cells with disrupted mitochondrial membrane. Primary neurons were incubated in DMEM medium containing 10 μM JC-1 at 37°C for 15 min, washed with PBS, and then transferred to a clear 12-well plate. JC-1 aggregate fluorescent emission was measured at 583 nm with an excitation wavelength of 526 nm; and JC-1 monomer fluorescence intensity was measured with excitation and emission wavelengths at 525 and 530 nm. Finally, the cells were mounted with the DakoCytomation fluorescent medium and visualized *via* fluorescence microscopy (Olympus Fluoview, Japan).

### Statistical analysis

All assays were performed on three separate occasions. Data are expressed as means ± S.D. All comparisons for parametric data were made using Student's test or one-way ANOVA followed by *post hoc* Turkey's test using the SPSS software (version 13.0: SPSS Inc., Chicago, IL, USA), GraphPad Prism 5 software (La Jolla, CA, USA) and Image J (National Institutes of Health, USA). P < 0.05 was considered statistically significant.

## SUPPLEMENTARY MATERIAL FIGURES


