# Supplementary material for: REST alleviates neurotoxic prion peptide-induced synaptic abnormalities, neurofibrillary degeneration and neuronal death partially via LRP6-mediated Wnt-β-catenin signaling
Source: Oncotarget. 2016 Feb 23;7(11):12035–52. doi: 10.18632/oncotarget.7640 (PMC4914267; doi:10.18632/oncotarget.7640)
Supplement: Supplementary file 1 [file oncotarget-07-12035-s001.pdf]

REST alleviates neurotoxic prion peptide-induced synaptic abnormalities, neurofibrillary degeneration and neuronal death partially *via* LRP6-mediated Wnt-β-catenin signaling

Supplementary Material

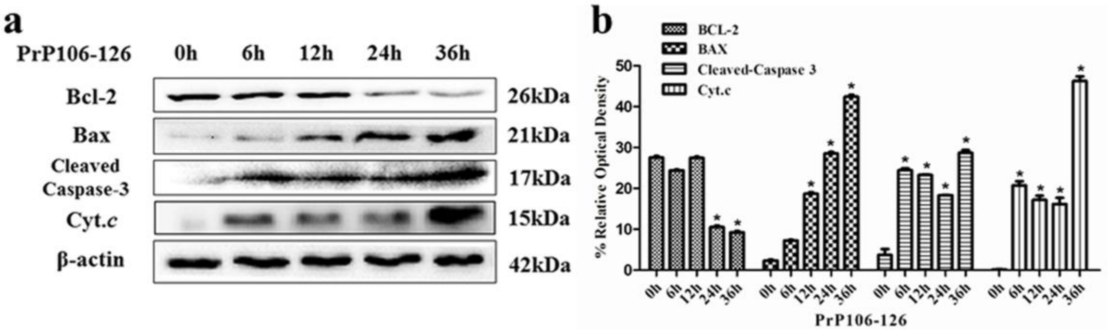

Supplementary Data Figure 1 (S1) PrP106-126 induces apoptosis-related protein changes in primary cultured cortical neurons (PCCN) (**a, b**). PCCN are treated with PrP106-126 and cytoplasmic fractions are collected at different time points (0h-36h) and processed for immunoblotting with anti- Bcl-2, BAX, cleaved-caspase 3 and cytochrome *c* antibodies. The protein levels are normalized to the expression of β-actin. Data are presented as mean±SD of triplicate experiments; $^{*}P < 0.05$ versus untreated cells.

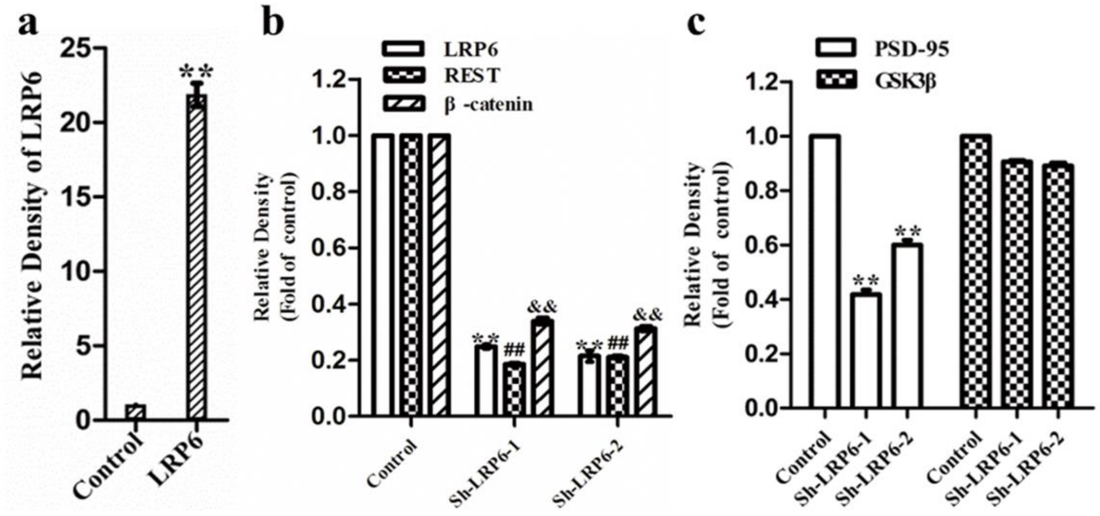

Supplementary Data Figure 2 (S2) Immunoblotting density analysis of LRP6 knockdown and associated proteins in figure 8. a Immunoblotting density of LRP6 in figure 8 d is normalized to β-actin expressed and expressed as ratio to the untreated control group. Data are presented as mean±SD of triplicate experiments. $^{**}P < 0.01$ versus the control group. **b** Immunoblotting density of protein in figure 8 f are normalized to β-actin expressed and expressed as ratio to the untreated control group. Data are presented as mean±SD of triplicate experiments. $^{**}P < 0.01$, $^{\#\#}P < 0.01$and $^{\&\&}P < 0.01$ versus the control group. **c** Immunoblotting density of protein in figure 8 g are normalized to GAPDH expressed and expressed as ratio to the untreated control group. Data are presented as mean±SD of triplicate experiments. $^{**}P < 0.01$ versus the control group.

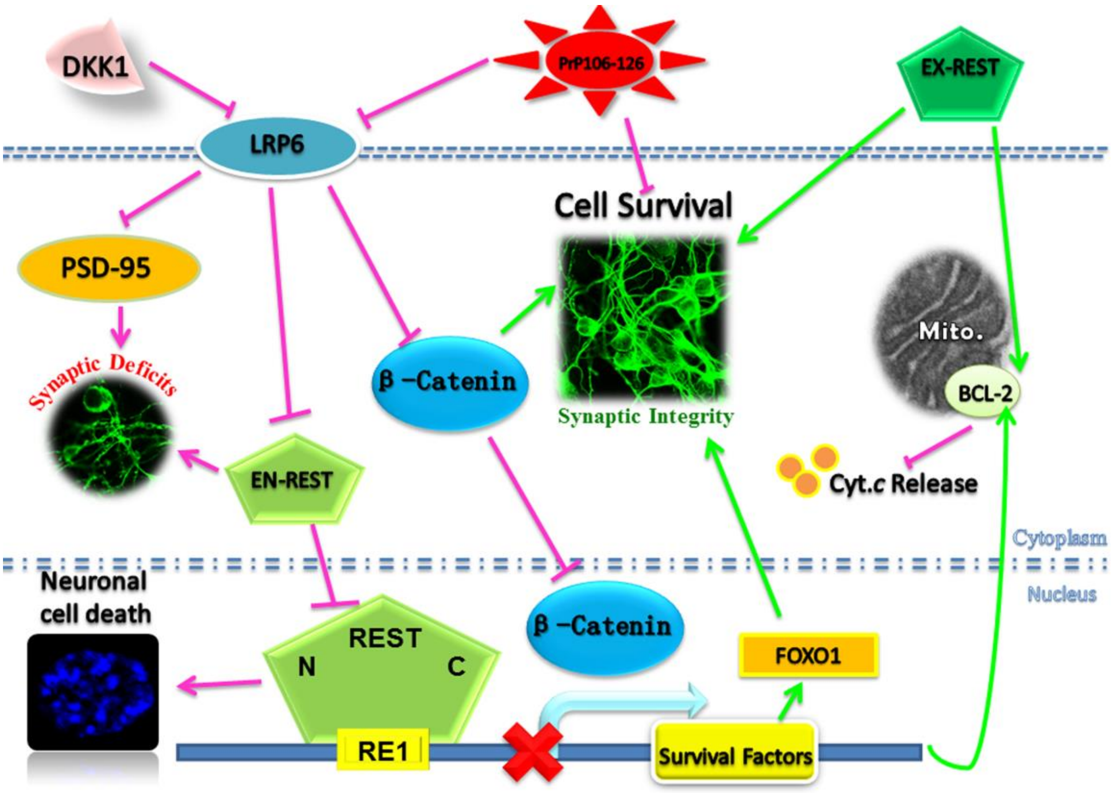

Supplementary Data Figure 3 (S3) Schematic signaling pathways for REST regulating PrP106-126-induced cellular morphological damage and neuronal cell death. After the stimulation of PrP106-126, endogenous REST transiently increases and translocates from cytoplasm to nucleus. Physiologically, LRP6 partially control the level of REST through the Wnt-signaling. Upon prolonged PrP106-126 incubation or inhibition by the Wnt signaling antagonist, DKK-1, LRP6 is suppressed and thereby results in a decrease in REST. Overexpressed REST acts as a neuroprotective regulator and contributes to neuronal survival by stabilizing the level of pro-survival protein FOXO1 and the mitochondrial outer membrane and inhibiting cytochrome c (Cyt.c) release from mitochondria to cytoplasm. LRP6-mediated Wnt-β-catenin signaling work cooperatively with REST to maintain the cellular morphological integrity by restoring the expression of PSD-95.
